# Combined Effects of Depression, Fatigue and Cardiovascular Dysfunction on Functional Dependence Over Seven Years in Early Parkinson's Disease

**DOI:** 10.1002/mdc3.70291

**Published:** 2025-09-12

**Authors:** Charlotte B. Stewart, Sahana Sathyanarayana, Victoria K. Foster, Robyn Iredale, Debra Galley, Jacopo Pasquini, Kirstie N. Anderson, Nicola Pavese, David Ledingham

**Affiliations:** ^1^ Clinical Ageing Research Unit, Newcastle University, Campus for Ageing and Vitality Newcastle upon Tyne United Kingdom; ^2^ Department of Clinical and Experimental Medicine University of Pisa Pisa Italy; ^3^ Regional Sleep Service, Newcastle upon Tyne, NHS Foundation Trust Newcastle upon Tyne United Kingdom; ^4^ Department of Nuclear Medicine and PET Centre Aarhus University Hospital Aarhus Denmark

**Keywords:** Parkinson's, PPMI, activities of daily living, functional dependence

## Abstract

**Background:**

Parkinson's disease (PD) is associated with both motor and non‐motor symptoms, which collectively impact activities of daily living (ADLs) and contribute to the loss of functional independence. There is a lack of understanding of how non‐motor symptoms drive this loss in independence.

**Objectives:**

This study aims to firstly implement a milestone‐based approach to determine the time‐points at which subjects with PD require help with specific tasks, using current gold‐standard scales. Secondly, we aimed to explore the influence of motor and non‐motor symptoms on the progression of functional dependence in individuals with early‐stage PD over a 7‐year period.

**Methods:**

Using data from 166 PD participants, obtained from the Parkinson's Progression Markers Initiative (PPMI), we determined the time taken to reach the first functional dependence “event” over 7 years of annual visits, using clinician‐ and patient‐reported outcomes, including the Schwab & England ADL scale and the MDS‐UPDRS Parts I and II. To determine the effect of non‐motor symptoms on functional dependence, linear mixed modelling was employed, adjusting for key motor variables, medication, age, sex and PD duration.

**Results:**

Depression, fatigue, and motor impairment emerged as significant influencers of functional dependence, with cardiovascular dysfunction nearing significance.

**Conclusions:**

We identified that, over time, both motor and non‐motor symptoms contributed to the decline in functional independence, with depression, fatigue and cardiovascular dysfunction playing a particularly prominent role. These findings highlight the importance of a comprehensive, multidisciplinary approach to PD management, addressing both motor and non‐motor symptoms to improve long‐term outcomes and quality of life.

Parkinson's disease (PD) is a progressive neurodegenerative disorder, characterized primarily by motor symptoms including; tremor, rigidity, bradykinesia, and postural instability.^1^ Patients with PD frequently experience a number of non‐motor symptoms including cognitive impairment, mood disorders, sleep disorders, and autonomic dysfunction.^2^ Both motor and non‐motor symptoms complicate PD management, and have a significant impact on patients’ activities of daily living (ADLs). ADLs are defined as the fundamental tasks required to live independently and be functionally independent.^3^ Basic ADLs, including washing, bathing, eating, and toileting, are essential for maintaining a patient's basic physical needs. Instrumental ADLs include more complex activities such as transportation, managing finances and communicating with others.^4^

Both motor and non‐motor symptoms typically worsen over time, patients increasingly find it harder to complete ADLs alone, leading to a loss of functional independence over time.^5^, ^6^, ^7^, ^8^, ^9^ Understanding the extent to which motor and non‐motor symptoms impact ADLs and functional dependence in *de novo* PD is crucial for developing comprehensive management strategies and treatments aimed at improving quality of life (QoL).^7^ Recently, Brumm and colleagues used a time‐to‐event‐based approach to determine milestone achievement in *de novo* PD subjects, highlighting the value in using this approach to determine PD progression.^10^ No study has yet used this approach to identify the specific time‐points at which individuals with PD reach their first functional dependence “event” for different ADLs.

There is a significant body of literature that recognizes the contribution of motor symptoms to functional dependence, however it is becoming increasingly clear that non‐motor symptoms also play an important role in an individual's ability to perform ADLs: recent studies have investigated the effect of non‐motor symptoms on ADLs in PD, as well as their impact on independent living and disability.^11^, ^12^, ^13^, ^14^ However, there is a gap in knowledge surrounding the key non‐motor predictors of functional dependence in the early stages of PD.

This study aims to explore the relative contribution of specific motor and non‐motor symptoms, experienced by individuals with early PD, on their ability to perform ADLs and hence their functional dependence over time. Using clinician and patient reported outcome data from the Parkinson's Progression Marker's Initiative (PPMI) study; we have proposed specific cut‐off scores for item‐responses based on when a patient will need the assistance of another person to carry out that task, as a marker of functional dependence. This study seeks to identify, which non‐motor symptoms are the most important contributors to functional dependence over time, and therein provide valuable insights for clinicians and caregivers, ultimately contributing to more effective and holistic management approaches for PD.

## Methods

### Study population

The study included 166 PD subjects, and 135 healthy control (HC) subjects retrieved from the PPMI database. PPMI is an ongoing longitudinal multicenter clinical study (study protocol and manuals are available at https://www.ppmi-info.org/study-design). PD subjects included in this analysis were sporadic, treatment naïve and diagnosed within the last 2 years of enrolment, aged ≥30. All participants had abnormal dopamine transporter–single photon emission computed tomography (DaT‐SPECT) confirming a loss in nigrostriatal dopaminergic neurons. HCs were ≥ 30 years of age with no cognitive impairment or first‐degree familial history of PD. Any HCs diagnosed with PD or dementia within the study period were excluded from analysis. The full list of exclusion criteria for the PPMI study can be viewed in the protocol (https://www.ppmi‐info.org/sites/default/files/docs/PPMI002_Clinical%20Protocol_AM3.2_30Jan2023_Final.pdf).

All PPMI sites received ethical approval, and all participants required written informed consent prior to study involvement. Data were obtained for participants who completed 7 years of follow‐up assessments, removing any participants with missing data during this time‐period to allow a comprehensive dataset of longitudinal data. We identified 166 PD participants with complete datasets for the 7‐year analysis, out of 231. For HCs, 5 years of follow‐up data for 135 participants was included in the analysis due to a small number of these individuals with MDS‐UPDRS data for 6 years (*n* = 18) and 7 years (*n* = 92).

Data used in the preparation of this article was obtained on August 25, 2023 from the Parkinson's Progression Markers Initiative (PPMI) database (www.ppmi-info.org/access-dataspecimens/download-data), RRID:SCR_006431. For up‐to‐date information on the study, visit www.ppmi-info.org. PPMI—a public‐private partnership—is funded by the Michael J. Fox Foundation for Parkinson's Research, and funding partners; including 4D Pharma, Abbvie, AcureX, Allergan, Amathus Therapeutics, Aligning Science Across Parkinson's, AskBio, Avid Radiopharmaceuticals, BIAL, BioArctic, Biogen, Biohaven, BioLegend, BlueRock Therapeutics, Bristol‐Myers Squibb, Calico Labs, Capsida Biotherapeutics, Celgene, Cerevel Therapeutics, Coave Therapeutics, DaCapo Brainscience, Denali, Edmond J. Safra Foundation, Eli Lilly, Gain Therapeutics, GE HealthCare, Genentech, GSK, Golub Capital, Handl Therapeutics, Insitro, Jazz Pharmaceuticals, Johnson & Johnson Innovative Medicine, Lundbeck, Merck, Meso Scale Discovery, Mission Therapeutics, Neurocrine Biosciences, Neuron23, Neuropore, Pfizer, Piramal, Prevail Therapeutics, Roche, Sanofi, Servier, Sun Pharma Advanced Research Company, Takeda, Teva, UCB, Vanqua Bio, Verily, Voyager v. 25MAR2024 Therapeutics, the Weston Family Foundation and Yumanity Therapeutics.

### Assessments/data collection

Functional dependence was measured using the Modified Schwab & England Activities of Daily Living (MSE‐ADL) scale. Clinician and patient‐reported outcomes of ADL impairment was assessed using the Movement Disorder Society‐Unified Parkinson's Disease Rating Scale (MDS‐UPDRS) Parts I‐II. Additional non‐motor impairment data were collected using specific questionnaires: Geriatric Depression Scale (GDS), State–Trait Anxiety Inventory (STAI), REM Sleep Behaviour Disorder Screening Questionnaire (RBD‐SQ), Epworth Sleepiness Scale (ESS), Montreal Cognitive Assessment (MoCA), Scales for Outcomes in Parkinson's‐Autonomic Dysfunction (SCOPA‐AUT), the University of Pennsylvania Smell Identification Test (UPSIT).

### Statistical analysis

Statistical analysis was conducted using SPSS version 29 for data analysis, and Microsoft Excel and RStudio was used for editing outputs. Baseline characteristics were compared between subjects using Chi‐square test for categorical variables and Mann–Whitney *U* tests for continuous, non‐parametric data.

The time‐to‐functional dependence analysis focused on specific items from the MDS‐UPDRS scale, particularly those where responses indicated the need for assistance from another person. The cut‐off for functional dependence varied depending on the item, determined by the point at which a patient first requires assistance in the activity. Thresholds were selected through a logical review of each item's descriptive anchors, guided by clinical judgement and consensus among study authors, including experienced PD clinicians. Although not statistically validated in this study, the approach was intended to maximize clinical interpretability and ensure that the milestones captured meaningful changes in independence. A similar method was employed by Ramsay and colleagues (2020), who applied clinically informed cut‐offs to the MDS‐UPDRS ADL items.^15^ Thresholds are outlined in Table S1. A cut‐off of <80% on the MSE‐ADL was employed to reflect the point at which individuals begin to require assistance with daily activities, supported by previous studies and clinical consensus.^15^

The frequencies of PD and HC subjects reporting non‐motor symptoms at baseline and 5 years were compared using Fisher's exact test and adjusted for multiple comparisons using Bonferroni correction. The percentage of subjects reporting non‐motor symptoms at baseline and 5 years follow‐up were compared between groups using McNemar's test, along with Bonferroni correction. Linear mixed modelling (LMM) was employed to determine non‐motor influencers of functional dependence (MSE‐ADL score < 80%) over 7 years in the PD cohort through their interactions with time; models were adjusted for sex, levodopa equivalent daily dose (LEDD) and age at PD diagnosis and months since PD diagnosis, as well as PD subtype. The LMM accounts for the fact that data is collected over time for each individual. Model fit was compared using the −2 Log Likelihood, Akaike Information Criterion (AIC) and Bayesian Information Criterion (BIC), with lower values indicating a better model fit. AIC and BIC balance model complexity and goodness‐of‐fit, correcting for model overfitting, and are commonly used for model selection in this context.^16^ Random intercepts were included to account for individual variability between participants; random slopes were also included if they improved model fit. The covariance structure giving the best model fit was also utilized. Fixed effect estimates were interpreted to assess the significance and direction of the non‐motor influencers of functional dependence over time. Spearman's rank correlations were used to determine the relationship between MSE‐ADL and SCOPA‐AUT‐cardiovascular scores.

## Results

### Baseline demographics and clinical characteristics

The baseline clinical features and demographics of patients with PD and healthy controls are presented in Table 1. The median age of PD symptom onset was 60.13 years with an interquartile range (IQR) of 14 years. The median age at PD diagnosis was 61.52 years (IQR = 14 years), and the median duration of PD at baseline was 4.13 months (IQR = 6 months). Among the presenting symptoms and signs, tremor was the most common, reported by 80.1% followed by bradykinesia (85.5%), rigidity (79.5%), and postural instability (5.4%). None of the patients were on PD treatment at baseline (0%) (Supplementary Table 2).

**TABLE 1:** Baseline demographics and clinical features of PD and HC subjects

	PD (*N* = 166)	HC (*N* = 135)	*P* (PD, HC)
Gender *N* (% f)	50 (30.1%)	52 (38.5%)	0.080*
Age	62.18 (14.47)	60.45 (13.73)	0.846*
Handedness *N* (% R)	139 (83.7%)	109 (80.7%)	0.791*
MDS‐UPDRS Part‐I	5 (5)	2 (4)	**<0.001** ^+^
MDS‐UPDRS Part‐II	5 (5)	0 (0)	**<0.001** ^+^
MDS‐UPDRS Part‐III	19 (11)	0 (2)	**<0.001** ^+^
MDS‐UPDRS Part‐IV	0 (0)	0 (0)	‐
MDS‐UPDRS Total	29 (18)	3 (6)	**<0.001** ^+^
Hoehn & Yahr	1 (1)	‐	‐
Schwab & England	95 (10)	‐	‐
Montreal cognitive assessment	28 (3)	28 (2)	**<0.001** ^+^
Epworth sleepiness scale	6 (5)	5.5 (5)	0.845^+^
RBD‐SQ	3 (4)	2 (3)	**<0.001** ^+^
Geriatric depression scale	2 (2)	1 (2)	**<0.001** ^+^
State–trait anxiety scale	64 (23)	52 (20)	**<0.001** ^+^
SCOPA‐AUT	8 (7)	5 (4)	**<0.001** ^+^

*Note*: Baseline demographics of Parkinson's disease (PD) and healthy control (HC) subjects. Data represented by median (interquartile range). Significance determined by Chi‐square tests* and Mann Whitney‐*U* tests+.

Abbreviations: RBD‐SQ‐ RBD Screening Questionnaire; SCOPA‐AUT‐ Scales for Outcomes in Parkinson's disease‐Autonomic.

### Time to first functional dependence event

Overall, 19 individuals (11.45%) with PD had already reached a functional dependence event in any category at baseline. Altogether, 65.06% of PD subjects reached a functional dependence event in any category, and 34.94% did not reach any event at all, over the 7‐year study (Table 2). No subjects reported an MSE‐ADL score below 80% at baseline. However, this number increased to 12 participants (7.23%) at the 7th follow‐up. Over the entire study period, 107 participants (35.54%) reported an MSE‐ADL score < 80% at some point over 7‐year period (Table 2).

**TABLE 2:** PD subjects reaching their first‐time functional dependence event over 7 years from baseline

	Time‐point										
ADL item	Outcome criteria	Baseline	1 Year	2 Year	3 Year	4 Year	5 Year	6 Year	7 Year	Total reached by 7 years	Not reached by 7 years
Eating	≥2	7	7	9	9	9	7	8	5	36.75%	63.25%
Dressing	≥2	8	6	13	6	11	13	9	6	43.37%	56.63%
Hygiene	≥2	1	0	1	1	2	4	5	6	12.05%	87.95%
Turning	≥2	0	1	2	1	8	10	12	10	26.51%	73.49%
Rising	≥2	7	10	7	8	7	14	16	10	47.59%	52.41%
Walking	≥3	1	2	0	0	3	0	6	14	15.66%	84.34%
Freezing	≥3	0	0	0	1	1	0	3	6	6.63%	93.37%
Constipation	=4	0	0	0	0	0	1	0	1	1.20%	98.80%
Swallowing	=4	0	0	0	0	0	1	0	0	0.60%	99.40%
Any New cases		19 (11.45%)	13 (7.83%)	19 (11.45%)	10 (6.02%)	10 (6.02%)	16 (9.64%)	13 (7.83%)	8 (4.82%)	65.06%	34.94%
*Cumulative total*		*19 (11.45%)*	*32 (19.28%)*	*51 (30.72%)*	*61 (36.75%)*	*71 (42.77%)*	*87 (52.41%)*	*100 (60.24%)*	*108 (65.06%)*		
Schwab and England score	< 80%	0	4	4	8	7	11	11	12	35.54%	64.46%

*Note*: The Number of PD subjects experiencing first‐time functional dependence (FD) over time on different items from the MDS‐UPDRS parts I‐II and Schwab & England. Red indicates a higher, and white a lower number of subjects experiencing FD.

### Non‐motor impairments in *de novo* PD

The prevalence of baseline non‐motor symptoms PD and HC subjects was compared. Cognitive impairment was present in two PD participants and no HCs (*P* = 0.503). Moderate to severe depression was reported by 2.42% of PD participants and 2.96% of HCs (*P* = 1.000). Moderate to severe apathy was not reported by any participants in either group at baseline (Table 3).

**TABLE 3:** Longitudinal changes in non‐motor symptoms in PD and HC subjects

Non‐motor symptom	Criteria	Baseline		BL	5 years		5 years	7 years
		PD (%)	HC (%)	*P*	PD (%)	HC (%)	*P*	PD (%)
Cognitive impairment	MDS‐UPDRS item 1.1 ≥ 3/MoCA <21	1.21	0.00	0.503*	5.45	0.00	0.005*	9.09
Depression	MDS‐UPDRS item 1.3 ≥ 3/GDS≥9	2.42	2.96	1.000*	5.45	0.74	0.026*	7.88
Apathy	MDS‐UPDRS item 1.5 ≥ 3	0.00	0.00	.	2.42	0.00	0.013*	3.03
EDS	MDS‐UPDRS item 1.8 ≥ 3/ESS ≥10	16.97	11.85	0.214	30.30	15.56	**0.003**	39.39
RBD	RBD‐SQ >6	20.00	7.41	**0.002**	26.67	5.19	**<0.001**	40.61
Anxiety	MDS‐UPDRS item 1.4 ≥ 3	1.21	0.74	1.000*	3.64	0.00	0.034*	2.42
Fatigue	MDS‐UPDRS item 1.13 ≥ 3	2.42	1.48	0.694*	7.88	0.74	0.004*	6.67
Gastrointestinal dysfunction	SCOPA‐AUT 1–7 ≥ 2/MDS‐UPDRS items 1.11, 2.2, 2.3 ≥ 3	52.12	20.00	**<0.001**	80.00	22.96	**<0.001**	83.64
Urinary dysfunction	SCOPA‐AUT 8–13 ≥ 2/MDS‐UPDRS item 1.10 ≥ 3	83.64	74.07	0.055	89.70	80.00	**0.018**	91.52
Cardiovascular dysfunction	SCOPA‐AUT 14–16 ≥ 2/MDS‐UPDRS item 1.12 ≥ 3	7.88	1.48	0.015*	20.61	2.22	<0.001*	26.67

*Note*: Percentage of Parkinson's disease (PD) and healthy control (HC) subjects reporting non‐motor symptoms in each assessment. Where two criteria were used for a symptom domain (eg, cognitive impairment), participants were considered positive if they met either criterion. *p*‐values determined using Chi‐square tests, except where cell counts were <5, in which case the Fisher's exact test was used (*). Values in bold indicate statistical significance *p* < 0.05.

Moderate to severe EDS was reported by 16.97% of PD participants and 11.85% of HCs (*P* = 0.214) at baseline. RBD symptoms were significantly more common in PD participants (20.00%) compared to HCs (7.41%) (*P* = 0.002). Moderate to severe anxiety was reported by two PD participants and one HC (*P* = 1.000). Moderate to severe fatigue was reported by 2.42% of PD and 1.48% of HC subjects (*P* = 0.694) (Table 3).

To evaluate autonomic symptoms, patient‐reported outcomes from the SCOPA‐AUT and MDS‐UPDRS were used. Gastrointestinal dysfunction was significantly more prevalent in PD participants (52.12%) compared to HCs (20.00%) at baseline (*p* < 0.001). Urinary dysfunction was reported by 83.64% of PD and 74.07% of HCs, approaching near significance (*P* = 0.055). Cardiovascular dysfunction was reported by 7.88% of PD participants and two HCs (1.48%) (*P* = 0.015) (Table 3).

After 5 years, all symptoms were significantly more prevalent in the PD cohort compared to HCs. However, only RBD (*P* = 0.0013), gastrointestinal (*P* < 0.001) and urinary symptoms (*P* < 0.001) were found to significantly increase over 5 years in PD. Gastrointestinal (*P* < 0.001) and urinary (*P* < 0.001) symptoms were also significantly increased in the HC cohort after 5 years, as well as EDS (*P* = 0.027). However, RBD symptoms significantly declined over the 5 years in the HC group (*P* < 0.001) (Table 3).

### Non‐motor influencers of functional dependence in PD over 7 years

To evaluate the impact of non‐motor variables on functional dependence (measured by a MSE‐ADL score < 80%), a LMM was used, adjusting for sex, age, LEDD, PD duration (in months), PD motor subtype (TD/PIGD‐I), and motor impairment (MDS‐UPDRS Part‐III score in the “on” state if applicable). The final model included depression, fatigue, cardiovascular dysfunction, and cognition. Although cognition was not found to be a significant predictor of functional dependence itself (*P* = 0.940), it was included in the final model due to its improvement to model fit following likelihood ratio tests in the hierarchal regression.

**Figure 1:**
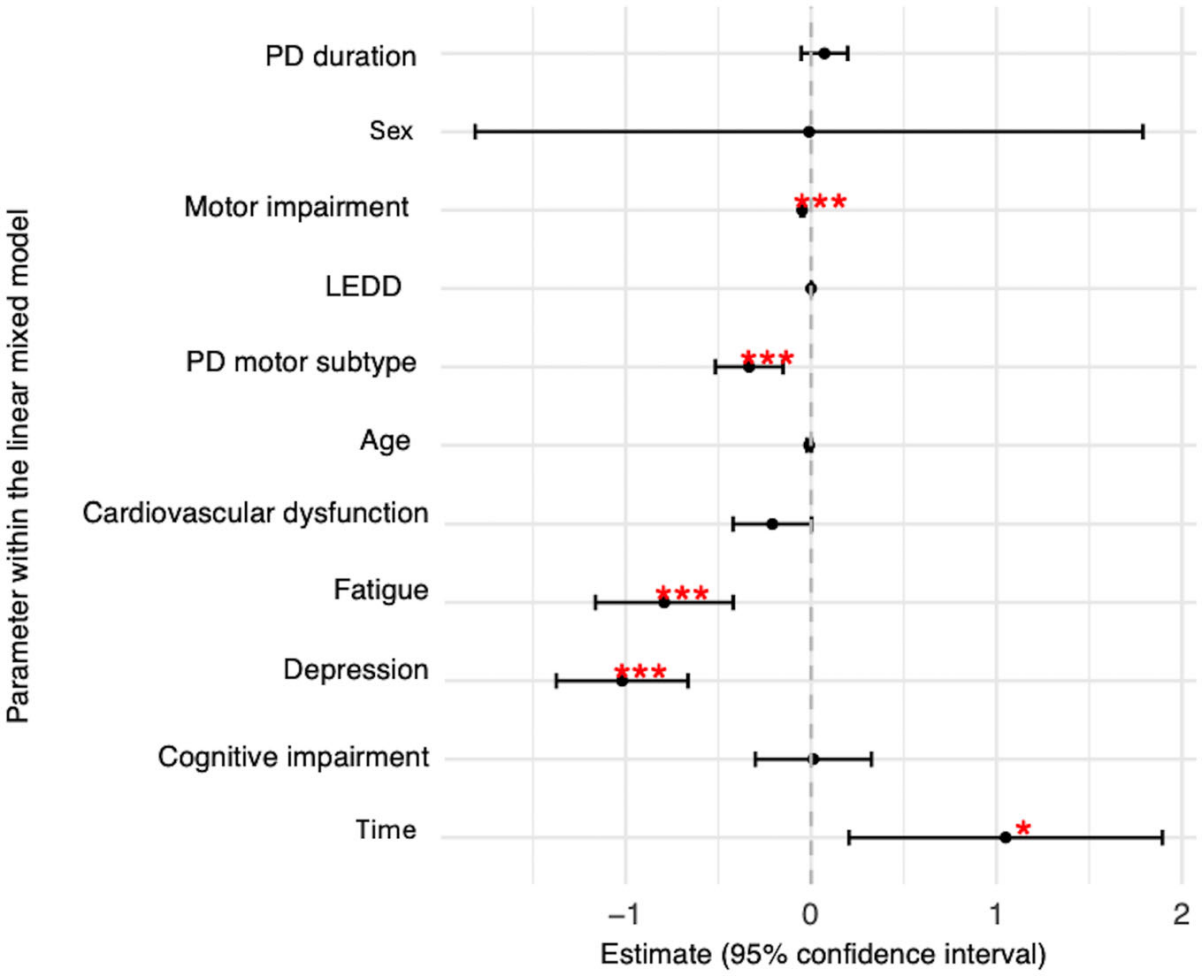
Estimated effects (95% confidence intervals) from the linear mixed model, assessing non‐motor symptoms on Schwab & England score over 7 years, adjusting for motor impairment, dopaminergic medication, and motor subtype. Significant contributors are marked with red asterisks (*P* < 0.05).

**Figure 2:**
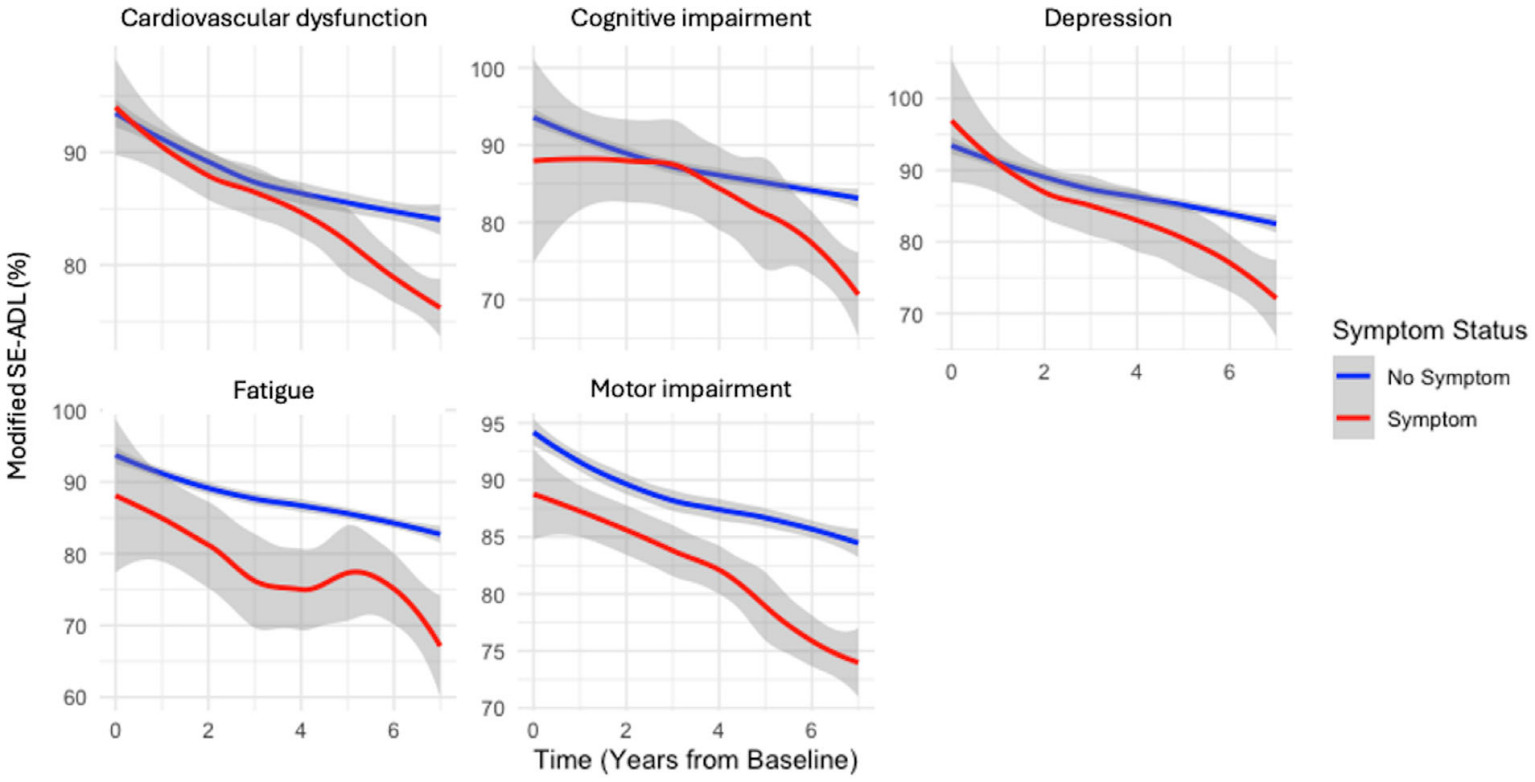
The individual effects of depression, fatigue and cardiovascular dysfunction on Schwab & England (SEADL) score over 7 years in PD. Estimated values determined from linear mixed modelling allowing for random slopes and intercepts with 95% confidence intervals.

Depression had the greatest influence on functional dependence across the 7‐year period (t = −5.64, *P* < 0.001), followed by fatigue (t = −4.18, *P* < 0.001) and cardiovascular dysfunction (t = −1.95, *P* = 0.052) (Fig. 1, Table S3). As expected, motor subtype and motor impairment (determined by the MDS‐UPDRS Part‐III) significantly contributed to functional dependence over the 7 years (Fig. 2). Cardiovascular sub‐score on the SCOPA‐AUT was negatively correlated with MSE‐ADL response (*P* = −0.388, *P* < 0.001), (Table S4; Fig. S1).

## Discussion

Overall, our study provides an in‐depth and comprehensive assessment of specific ADLs and their impact on functional dependence through the interpretation of scores from current gold‐standard measures of PD severity. In a recent study, Brumm and colleagues implemented time‐to‐event analysis to determine clinically meaningful milestones of PD progression.^10^ Adopting this milestone‐based approach, this paper specifically investigates the time‐points at which PD participants become functionally dependent for specific tasks.

Most participants (65.06%) reached a functional dependence milestone by the end of the 7 years, yet, a significant portion of the study population remained independent throughout the observation period. The MSE‐ADL score was ≥80% in all participants at baseline, with only twelve scoring <80% at 7 years. Among the ADLs, the functional dependence milestones for eating, dressing, and rising from a chair were reached the earliest, suggesting these tasks are the most common initial challenges to independence. In contrast, milestones for hygiene, turning in bed, swallowing, walking, and freezing were typically reached later, generally from 4 years onwards. The timing of functional dependence in eating and hygiene tasks varied depending on the Hoehn & Yahr stage, highlighting stage‐specific differences in disease progression.

Only two participants reached the constipation milestone, likely because this level of dependence is more severe and less common than others like bathing and dressing. Because constipation was the only non‐motor symptom with an outcome for functional dependence in the MDS‐UPDRS, this study highlights a need for this outcome in other non‐motor symptoms, such as cognition, depression, anxiety, sleep and autonomic symptoms. This would allow a comprehensive investigation into milestone achievement for functional dependence, allowing a better understanding of non‐motor ADL burden.

This study builds upon recent work by Brumm and colleagues,^10^ in which they use a milestone‐based approach for a more clinically meaningful assessment of PD progression. In fact, our study uses a similar approach to explore functional dependence milestones in more detail. However, to comprehensively investigate loss of independence, we used patient reported outcomes for functional dependence in different daily tasks, as opposed to the MSE‐ADL scale only.

The LMM further underscores the significant impact of non‐motor symptoms on the progression of functional dependence in patients with de novo PD over a 7‐year period. The results demonstrate that non‐motor symptoms including depression, fatigue, and cardiovascular dysfunction may directly contribute to functional dependence, from early to more advanced stages of PD, independently of motor symptoms. Furthermore, these non‐motor symptoms may interact to accelerate functional decline over time, though, further research is required to clarify their combined effects. Overall, these findings support previous research highlighting the key role of non‐motor symptoms on independence in PD.^3^, ^6^

In PD, the presence and worsening of cardiovascular symptoms, depression, and fatigue significantly impacts functional independence in daily activities. Depression is a prevalent non‐motor symptom of PD and closely correlates with physical limitations and subsequent functional decline, as identified in previous studies.^17^, ^18^, ^19^ The neurochemical changes in PD predispose individuals to mood disorders.^20^ These changes include dysfunction in dopamine, serotonin, and norepinephrine systems, which are heavily involved in mood regulation.^21^, ^22^ Depression in PD not only contributes to a lack of motivation and interest in daily activities but may also affect cognitive function and reduce the ability to plan and execute tasks. Depression may lower an individual's energy levels, exacerbating fatigue, and creating a sense of helplessness, all of which can diminish independence.^23^, ^24^ This mental health component may create a negative feedback loop in which reduced activity and social isolation can reinforce depressive symptoms, further impairing daily functioning.

Fatigue, both physical and mental, is one of the most common and debilitating symptoms in PD, and in this study, substantially contributes to functional dependence, which has also been highlighted by previous research.^5^, ^25^ Furthermore, fatigue can be associated with dopaminergic medication side effects, sleep disturbances, and the effort required to manage motor symptoms like bradykinesia. Unlike simple tiredness, fatigue in PD is pervasive, and may not always improve with rest, limiting the ability to complete even simple tasks consistently.^25^, ^26^ Additionally, the presence of fatigue often leads to task avoidance and a reduced capacity to maintain daily routines, significantly lowering QoL and independence.^27^

This study also identified cardiovascular dysfunction to be a borderline significant factor in the loss of functional independence in PD. Alongside depression and fatigue symptoms, the contribution of cardiovascular dysfunction on functional dependence may be overshadowed, as there is a significant relationship between functional dependence and cardiovascular decline, shown in Figure S1. This supports previous findings of cardiovascular decline to be a risk marker for functional impairment in PD.^28^ While several pathophysiological mechanisms have been proposed to explain the link between cardiovascular dysfunction, mood disturbances, and fatigue in PD, it is equally important to consider the potential for secondary cardiovascular deconditioning. Impairments in mobility and the presence of depression—both common in PD—can lead to reduced physical activity, which in turn may contribute to diminished cardiovascular fitness and autonomic responsiveness. This bidirectional relationship suggests that cardiovascular abnormalities may not always be a primary manifestation of PD, but could also arise because of prolonged inactivity and psychosocial burden. Recognizing this interplay is essential for interpreting symptom progression and for designing interventions that address both primary neurodegenerative changes and secondary lifestyle‐related effects.

PD is often accompanied by autonomic dysfunction, leading to abnormalities in heart rate and blood pressure regulation through mechanisms such as cardiac sympathetic denervation and reduced norepinephrine transmission.^29^ These changes contribute to cardiovascular dysregulation, including orthostatic hypotension—a condition affecting approximately 30–40% of individuals with PD. Orthostatic hypotension can lead to cerebral hypoperfusion, particularly in the frontal and limbic regions, potentially exacerbating mental fatigue and depressive symptoms.^30^ Importantly, norepinephrine plays a dual role in both peripheral autonomic control and central nervous system function. Peripherally, it is essential for maintaining vascular tone and blood pressure stability. Centrally, norepinephrine modulates arousal, attention, and mood via projections from the locus coeruleus to the prefrontal cortex, limbic system, and brainstem nuclei. Thus, degeneration of noradrenergic neurons in PD may simultaneously impair cardiovascular reflexes and disrupt neural circuits involved in motivation and affective regulation. This shared neurochemical pathway may partially explain the co‐occurrence of orthostatic symptoms, fatigue, and depression in PD.^31^

As well as directly influencing functional decline in PD, it is possible that cardiovascular symptoms may also interact with depressive symptoms to drive functional decline over the 7 years. To prevent overcomplicating the model and introducing multicollinearity, three‐way interactions with cardiovascular and depressive symptoms with time were avoided. However, there was a significant positive correlation between SCOPA‐AUT cardiovascular score and GDS from 1 year follow‐up, onwards, as well as with MSE‐ADL at 6 and 7 years (Fig. S1), suggesting that there are associations between mood and cardiovascular function, which we have identified already as individual contributors of functional decline. This would support recent findings which have identified that longitudinal changes in cardiovascular function were able to predict increasing ADL impairment,^18^ as well as a higher degree of functional dependence,^5^ through its effect on depressive symptoms. The reason for this association may be that cardiovascular symptoms can lead to repeated falls, anxiety, subsequent physical inactivity and fatigue, leading to depression and loss of independence. Furthermore, there is evidence that antidepressant medication may increase the risk of orthostatic hypotension,^32^ further contributing to this interaction between cardiovascular and depression. Future research should further explore the effects of the relationship between depression and cardiovascular symptoms on functional dependence over time in PD in a more complex model with multiple levels of interactions.

In conclusion, cardiovascular dysfunction, depression, and fatigue in PD can create physical, emotional, and cognitive barriers to functional independence. These factors may not be entirely isolated but may reinforce one another, creating a cycle of reduced mobility, motivation, and energy, that severely limits the ability to engage in daily activities independently. Addressing these symptoms in combination, rather than in isolation, is crucial for improving quality of life and enhancing functional outcomes in PD. Clinically, this highlights the importance of screening for all three of these non‐motor symptoms, when only one is present. For example, a PD patient with orthostatic hypotension should also be evaluated for depressive symptoms and fatigue.^33^ Treatment of these symptoms within this cluster early may prevent or delay functional decline.

The effect of these symptoms on independence over time underscores the need for integrated, multidisciplinary interventions that address cardiovascular dysfunction, fatigue, and depression concurrently to mitigate the decline in functional independence in PD. These findings also support a growing emphasis on tailored, symptom‐specific PD interventions. Blood pressure medication for orthostatic hypotension like fludrocortisone and droxidopa^34^ could potentially reduce depressive symptoms and fatigue through shared pathways as described earlier in this paper, as well as through increased confidence when moving around. Simultaneously, addressing depressive symptoms with medications like selective serotonin reuptake inhibitors (SSRIs) and psychotherapy^35^ could potentially improve motivation, mobility, autonomic regulation, and in turn, cardiovascular function.^36^, ^37^ However, clinicians should be aware of the potential added risk of orthostatic hypotension and select treatment plans accordingly.^38^

A limitation of using the MDS‐UPDRS ADL assessments for our study is that not every ADL item includes an outcome option for functional dependence. Developing more comprehensive patient‐reported ADL outcomes by addressing independence would provide a more clinically meaningful assessment, ultimately facilitating more effective management plans. It is also worth noting the subjective nature of the assessments; with questionnaires being subject to inter‐rater variability within and between clinical sites. Another limitation of this study is that the impact of medications for non‐motor symptoms over the study period was not addressed due to time limitations. Future studies should consider incorporating data on the use of medications for these symptoms, including antidepressants and cardiovascular agents, which may have interconnected effects on other symptoms.

This study highlights the significant role of both motor and non‐motor symptoms, particularly depression, fatigue, and cardiovascular dysfunction, in driving functional dependence in early PD. While motor symptoms like postural instability contribute early on, non‐motor symptoms progressively accelerate decline, as neurodegeneration becomes more widespread over time. The findings underscore the need for a multidisciplinary approach to PD treatment, addressing both motor and non‐motor symptoms to slow dependence. Targeted interventions, such as psychological support, motivational coaching, and exercise programs, could reduce the progression of functional dependence, improving patient outcomes and easing the burden on caregivers and healthcare systems.

## Author Roles

(1). Research Project: A. Conception, B. Data collection; (2) Statistical Analysis: A. Design, B. Execution, C. Review and Critique; (3) Manuscript Preparation: A. Writing the First Draft, B. Review and Critique.

C.B.S.: 1A, 1B, 2A, 2B, 3A

S.S.: 1B, 2C, 3B

V.F.: 1B, 3B

R.I.: 1B, 2B, 3B

D.G.: 1B, 3B

J.P.: 2C, 3C

K.N.A.: 2C, 3C

N.P.: 1A, 1B, 1A, 2C, 3A

D.L.: 1A, 1B, 1A, 2C, 3A

## Disclosures

**Ethical Compliance Statement:** The PPMI study was conducted across multiple international sites, with ethical approval secured independently at each location and informed consent obtained from all participants. The research complied with the ethical principles of the Declaration of Helsinki and adhered to Good Clinical Practice guidelines, following review and approval by local ethics committees. We acknowledge the Journal's policies on ethical publication and confirm that this work fully complies with those guidelines.

**Funding Sources and Conflicts of Interest:** No specific funding was received for this work. The authors declare that there are no conflicts of interest relevant to this work.

**Financial Disclosures for the Previous 12 Months:** NP is a member the following Advisory Boards: Britannia, Boston Scientific, Benevolent AI, Roche, Abbvie, Teva. He is a recipient of the following honoraria: Britannia, Abbvie, GE Healthcare, Boston Scientific, and the following grants: Independent Research Fund Denmark, Danish Parkinson's disease Association, Parkinson's UK, Center of Excellence in Neurodegeneration (CoEN) network award, GE Healthcare Grant, Multiple System Atrophy Trust, Weston Brain Institute, EU Joint Program Neurodegenerative Disease Research (JPND), EU Horizon 2020 research, the Michael J. Fox Foundation and F. Hoffmann‐La Roche. DL: is employed by Newcastle Upon Tyne NHS Foundation Trust and Newcastle University. He is a recipient of the following honoraria: Dystonia Europe, Medtronic, Merz. He has received royalties from Oxford University Press, and grants from Dystonia Europe and the Michael J. Fox Foundation. He has also received travel grants from Ipsen, Bial, Medtronic and Boston Scientific. KNA has received financial support to attend educational meetings, speaker fees, and medical advisory boards from UCB, Esai, Stowood Scientific Instruments, Jazz Pharmaceuticals, Idorsia, Bioprojet, Janssen Cilag, Oxevision, Somnomed, Resmed, REMserve, Flynn pharma. She has also received academic funding from the Academy of Health Sciences, the Health Foundation, the Michael J. Fox Foundation, BBSRC and Unilever, MRC, UKRI and the Circadian Mental Health Network.

## Supporting information


**Supplementary Figure S1.** Scatter plot of the Modified Schwab & England (MSE‐ADL) score at 7 years follow‐up versus cardiovascular sub‐score in the Scales for Outcomes in Parkinson's Disease‐Autonomic (SCOPA‐AUT) score in Parkinson's disease subjects. The dashed lines indicate the 95% confidence intervals. Higher cardiovascular SCOPA‐AUT scores at 7 years are associated with a decline in SEADL scores, suggesting greater functional dependence with increasing cardiovascular dysfunction. The R^2^ value of 0.134 indicates the proportion of variance in SEADL scores explained by cardiovascular dysfunction. The two variables show a significant negative correlation (Spearman's Rank; ρ = −0.388, *P* < 0.001).

**Supplementary TABLE S1.** Different patient‐reported outcomes of activities of daily living from the Movement Disorder Society‐Unified Parkinson's Disease Rating Scale (MDS‐UPDRS) Part 1 (Non‐Motor Aspects of Experiences of Daily Living) and Part 2 (Motor Aspects of Experiences of Daily Living) and the cut‐off scores which imply functional dependence for each item. Both parts 1 and 2 range from 1 to 13 and assess common PD symptoms in the past week.
**Supplementary TABLE S2.** PD‐specific clinical features at baseline, including age at motor symptom onset, age at PD diagnosis and duration of PD since diagnosis. Presenting symptom (tremor, rigidity, bradykinesia, and postural instability) derived from Movement Disorder Society‐Unified Parkinson's Disease Rating Scale (MDS‐UPDRS) Part 3 results. Values represented as median (interquartile range) or *N* (%).
**Supplementary TABLE S3.** Estimates from the linear mixed model assessing the impact of non‐motor symptoms, motor impairment, and clinical covariates on functional dependence in Parkinson's disease over 7 years. The table presents the parameter estimates, standard errors, degrees of freedom (df), t‐values, *P*‐values, and 95% confidence intervals for each parameter. Significant effects (bold) are observed for time alone, depression, fatigue, motor subtype, and motor impairment, indicating their influence on the progression of functional dependence. Cardiovascular dysfunction approaches significance (*P* = 0.052), suggesting a potential role in long‐term outcomes.
**Supplementary TABLE S4.** Correlation coefficients and significance values for the association between cardiovascular sub‐score in the Scales for Outcomes in Parkinson's Disease‐Autonomic (SCOPA‐AUT), and the Modified Schwab & England (MSE‐ADL) score and Geriatric depression scale (GDS) score at baseline to 7 years follow‐up in Parkinson's disease subjects. Alpha value *P* = 0.05.

## Data Availability

The data that support the findings of this study are openly available in m the Parkinson's Progression Markers Initiative database at http://www.ppmi-info.org/access-data-specimens/download-data, reference number RRID:SCR_006431.
