# Supplementary figures and images for: Combined Effects of Depression, Fatigue and Cardiovascular Dysfunction on Functional Dependence Over Seven Years in Early Parkinson's Disease
Source: Mov Disord Clin Pract. 2025 Sep 12;13(2):399–409. doi: 10.1002/mdc3.70291 (PMC12911524; doi:10.1002/mdc3.70291)

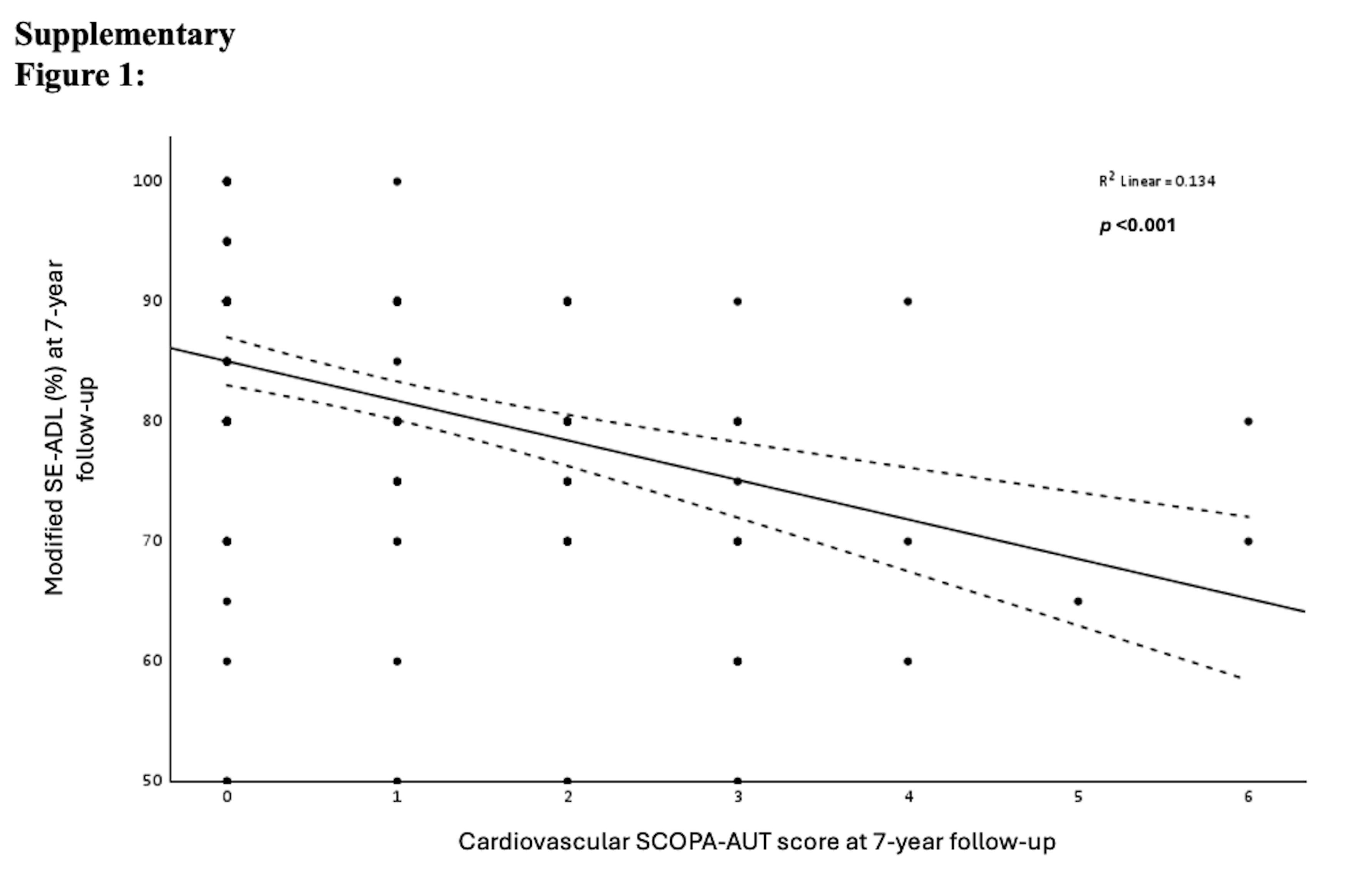

Supplement: Supplementary file 1 — Supplementary Figure S1. Scatter plot of the Modified Schwab & England (MSE‐ADL) score at 7 years follow‐up versus cardiovascular sub‐score in the Scales for Outcomes in Parkinson's Disease‐Autonomic (SCOPA‐AUT) score in Parkinson's disease subjects. The dashed lines indicate the 95% confidence intervals. Higher cardiovascular SCOPA‐AUT scores at 7 years are associated with a decline in SEADL scores, suggesting greater functional dependence with increasing cardiovascular dysfunction. The R2 value of 0.134 indicates the proportion of variance in SEADL scores explained by cardiovascular dysfunction. The two variables show a significant negative correlation (Spearman's Rank; ρ = −0.388, P < 0.001). [file MDC3-13-399-s002.png]
