# Supplementary material for: Combined Effects of Depression, Fatigue and Cardiovascular Dysfunction on Functional Dependence Over Seven Years in Early Parkinson's Disease
Source: Mov Disord Clin Pract. 2025 Sep 12;13(2):399–409. doi: 10.1002/mdc3.70291 (PMC12911524; doi:10.1002/mdc3.70291)
Supplement: Supplementary file 2 — Supplementary TABLE S1. Different patient‐reported outcomes of activities of daily living from the Movement Disorder Society‐Unified Parkinson's Disease Rating Scale (MDS‐UPDRS) Part 1 (Non‐Motor Aspects of Experiences of Daily Living) and Part 2 (Motor Aspects of Experiences of Daily Living) and the cut‐off scores which imply functional dependence for each item. Both parts 1 and 2 range from 1 to 13 and assess common PD symptoms in the past week. Supplementary TABLE S2. PD‐specific clinical features at baseline, including age at motor symptom onset, age at PD diagnosis and duration of PD since diagnosis. Presenting symptom (tremor, rigidity, bradykinesia, and postural instability) derived from Movement Disorder Society‐Unified Parkinson's Disease Rating Scale (MDS‐UPDRS) Part 3 results. Values represented as median (interquartile range) or N (%). Supplementary TABLE S3. Estimates from the linear mixed model assessing the impact of non‐motor symptoms, motor impairment, and clinical covariates on functional dependence in Parkinson's disease over 7 years. The table presents the parameter estimates, standard errors, degrees of freedom (df), t‐values, P‐values, and 95% confidence intervals for each parameter. Significant effects (bold) are observed for time alone, depression, fatigue, motor subtype, and motor impairment, indicating their influence on the progression of functional dependence. Cardiovascular dysfunction approaches significance (P = 0.052), suggesting a potential role in long‐term outcomes. Supplementary TABLE S4. Correlation coefficients and significance values for the association between cardiovascular sub‐score in the Scales for Outcomes in Parkinson's Disease‐Autonomic (SCOPA‐AUT), and the Modified Schwab & England (MSE‐ADL) score and Geriatric depression scale (GDS) score at baseline to 7 years follow‐up in Parkinson's disease subjects. Alpha value P = 0.05. [file MDC3-13-399-s001.docx]

# Supplementary Material for Review and Publication

### Supplementary Table 1: Criteria used for investigating functional dependence milestones in the MDS-UPDRS parts 1 and 2

| Motor/non-motor | Activity of daily living name | Item on MDS-UPDRS | Criteria for reaching functional dependence |
| --- | --- | --- | --- |
| Non-motor | Constipation problems | Item 1.11 | Score of 4 |
| Motor | Chewing and swallowing | Item 2.3 | Score of 4 |
|  | Eating tasks | Item 2.4 | Score ≥ 2 |
|  | Dressing | Item 2.5 | Score ≥ 2 |
|  | Hygiene | Item 2.6 | Score ≥ 2 |
|  | Turning in bed | Item 2.9 | Score ≥ 2 |
|  | Getting out of a bed, car, or deep chair | Item 2.11 | Score ≥ 2 |
|  | Walking and balance | Item 2.12 | Score ≥ 3 |
|  | Freezing | Item 2.13 | Score ≥ 3 |

### Supplementary Table 2: PD-specific clinical features at baseline

| ​PD symptoms at Baseline | PD (N=166)​ |
| --- | --- |
| Age at symptom onset (years)​ | 60.13 (14)​ |
| Age at PD diagnosis (years)​ | 61.52 (14)​ |
| PD duration (months)​ | 4.13 (6)​ |
| Presenting symptom​ | ​ |
| *Tremor ​* | 133 (80.1%)​ |
| *Rigidity ​* | 132 (79.5%)​ |
| *Bradykinesia​* | 142 (85.5%)​ |
| *Postural instability​* | 9 (5.4%)​ |
| On PD treatment​ | 0 (0%)​ |

**Supplementary Table 3: Estimates for the linear mixed model of functional dependence in PD over seven years**

| **Parameter** | **Estimate** | **Std. Error** | **df** | **t-value** | ***p*** | **Lower 95% CI** | **Upper 95% CI** |
| --- | --- | --- | --- | --- | --- | --- | --- |
| Intercept | 91.723 | 0.914 | 189.982 | 100.39 | **<.001** | 89.921 | 93.525 |
| Time | 1.049 | 0.431 | 1245.937 | 2.44 | **0.015** | 0.204 | 1.894 |
| Cognition x time | 0.012 | 0.159 | 1239.688 | 0.08 | 0.940 | -0.301 | 0.325 |
| Depression x time | -1.019 | 0.181 | 1269.889 | -5.64 | **<.001** | -1.374 | -0.664 |
| Fatigue x time | -0.792 | 0.189 | 1279.869 | -4.18 | **<.001** | -1.163 | -0.42 |
| Cardiovascular dysfunction x time | -0.209 | 0.108 | 1254.039 | -1.95 | 0.052 | -0.421 | 0.002 |
| Age x time | -0.011 | 0.006 | 1187.893 | -1.85 | 0.065 | -0.023 | 0.001 |
| PIGD subtype x time | -0.334 | 0.093 | 1276.305 | -3.59 | **<.001** | -0.516 | -0.151 |
| LEDD x time | 0 | 0 | 1281.641 | 1.00 | 0.316 | 0 | 0 |
| Motor impairment x time | -0.048 | 0.004 | 1302.313 | -11.55 | **<.001** | -0.056 | -0.04 |
| Sex | -0.011 | 0.912 | 161.511 | -0.01 | 0.990 | -1.812 | 1.789 |
| PD duration at BL (months) | 0.072 | 0.063 | 159.111 | 1.14 | 0.256 | -0.053 | 0.197 |

### Supplementary Table 4: Correlations of SE-ADL and GDS score by cardiovascular SCOPA-AUT score at baseline to seven years follow-up in PD participants

| Time-point | Spearman's Rho  SE-ADL x SCOPA-CV | | Spearman's Rho  GDS x SCOPA-CV | |
| --- | --- | --- | --- | --- |
|  | Correlation coefficient | Sig. (*p*) | Correlation coefficient | Sig. (*p*) |
| Baseline | 0.088 | 0.262 | 0.07 | 0.370 |
| 1 Year | -0.031 | 0.689 | 0.273 | < 0.001 |
| 2 Year | -0.132 | 0.091 | 0.181 | 0.020 |
| 3 Year | -0.129 | 0.098 | 0.216 | 0.005 |
| 4 Year | -0.107 | 0.170 | 0.263 | 0.001 |
| 5 Year | -0.165 | 0.034 | 0.344 | < 0.001 |
| 6 Year | -0.237 | 0.002 | 0.308 | < 0.001 |
| 7 Year | -0.388 | < 0.001 | 0.391 | < 0.001 |
